# Supplementary figures and images for: The long-run effects of secondary school track assignment
Source: PLoS One. 2019 Oct 25;14(10):e0215493. doi: 10.1371/journal.pone.0215493 (PMC6814234; doi:10.1371/journal.pone.0215493)

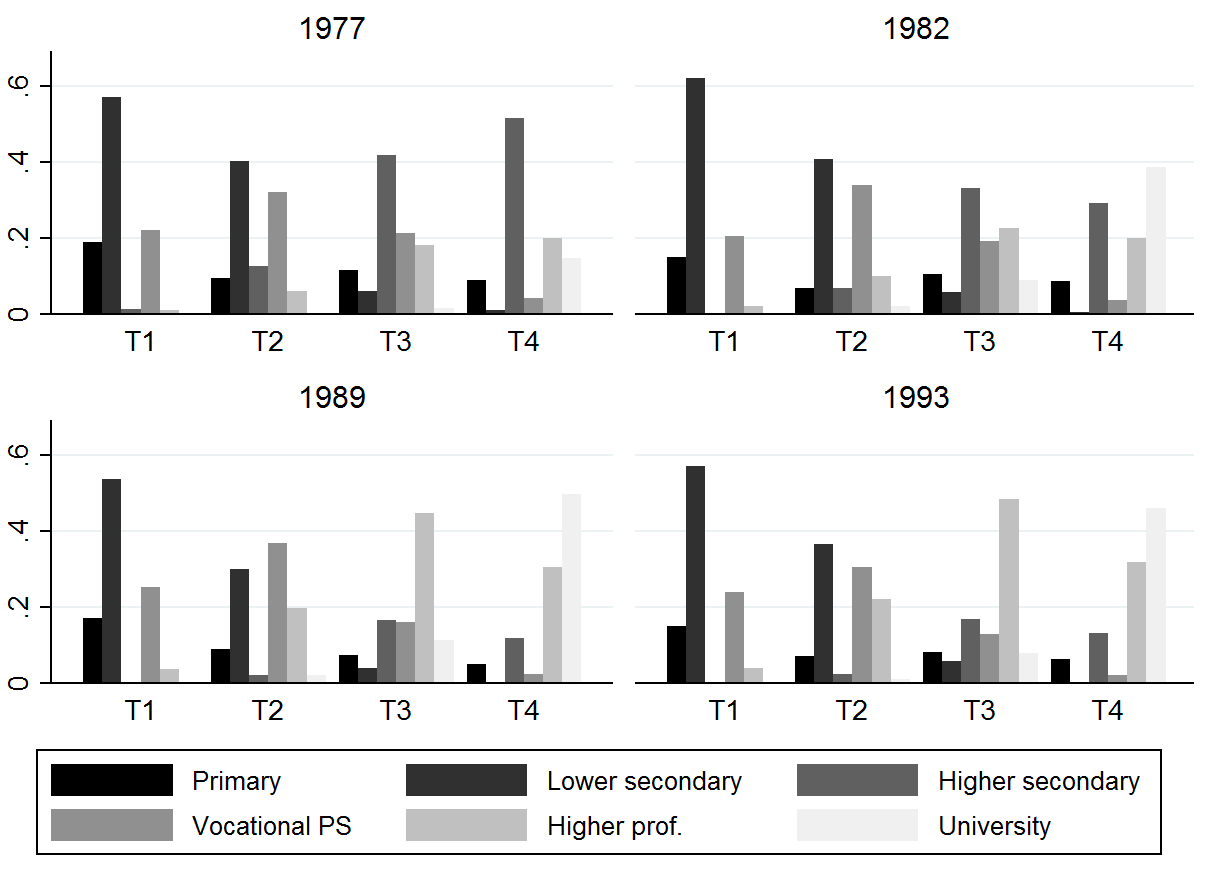

Supplement: S1 Fig — Final educational attainment across tracks that the students are (initially) assigned to in secondary school, separately for each cohort. (TIF) [file pone.0215493.s001.tif]

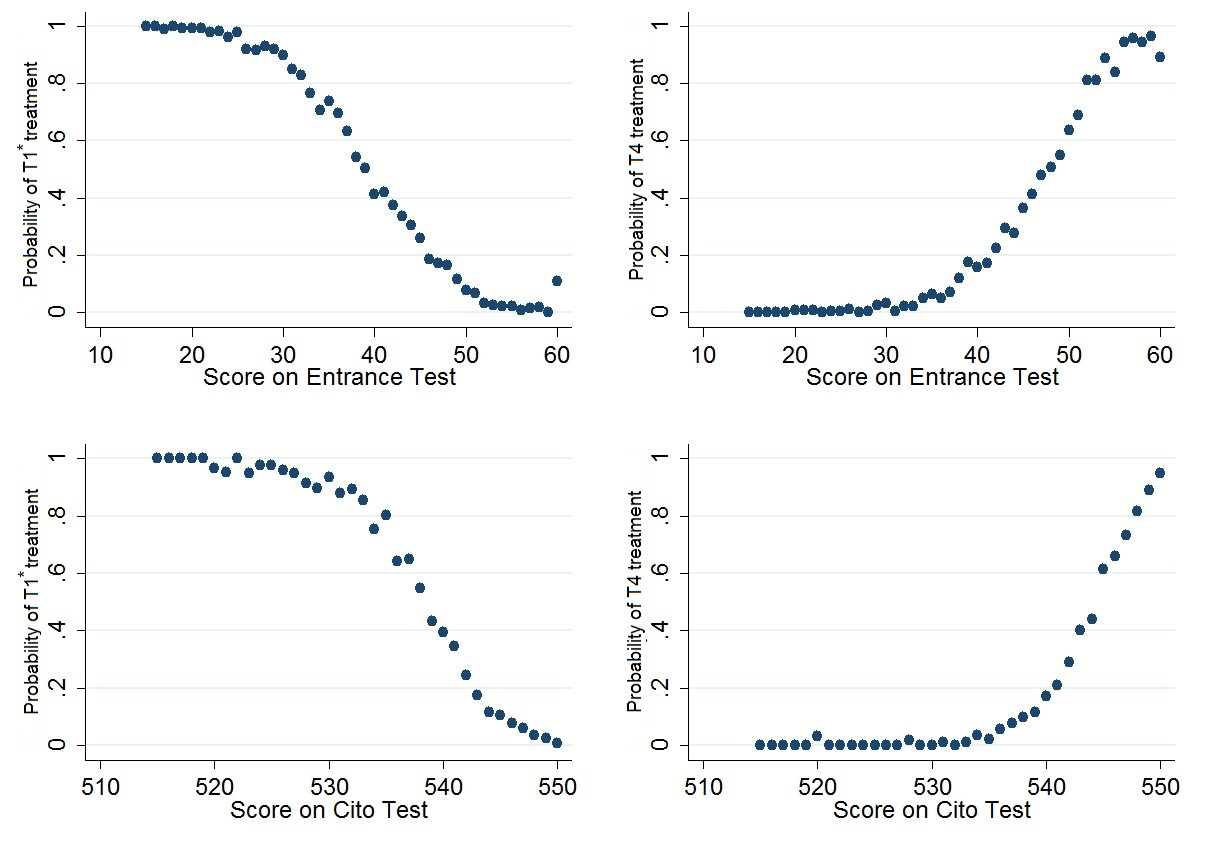

Supplement: S2 Fig — Shares of students that are sent to either the merged vocational track (left; labeled T1*) or the top track T4 (right), when using the low-stakes Entrance Test as the forcing variable (top) and when using the high-stakes Cito test as the forcing variable (bottom). All students entered secondary education in 1999. (TIF) [file pone.0215493.s002.tif]

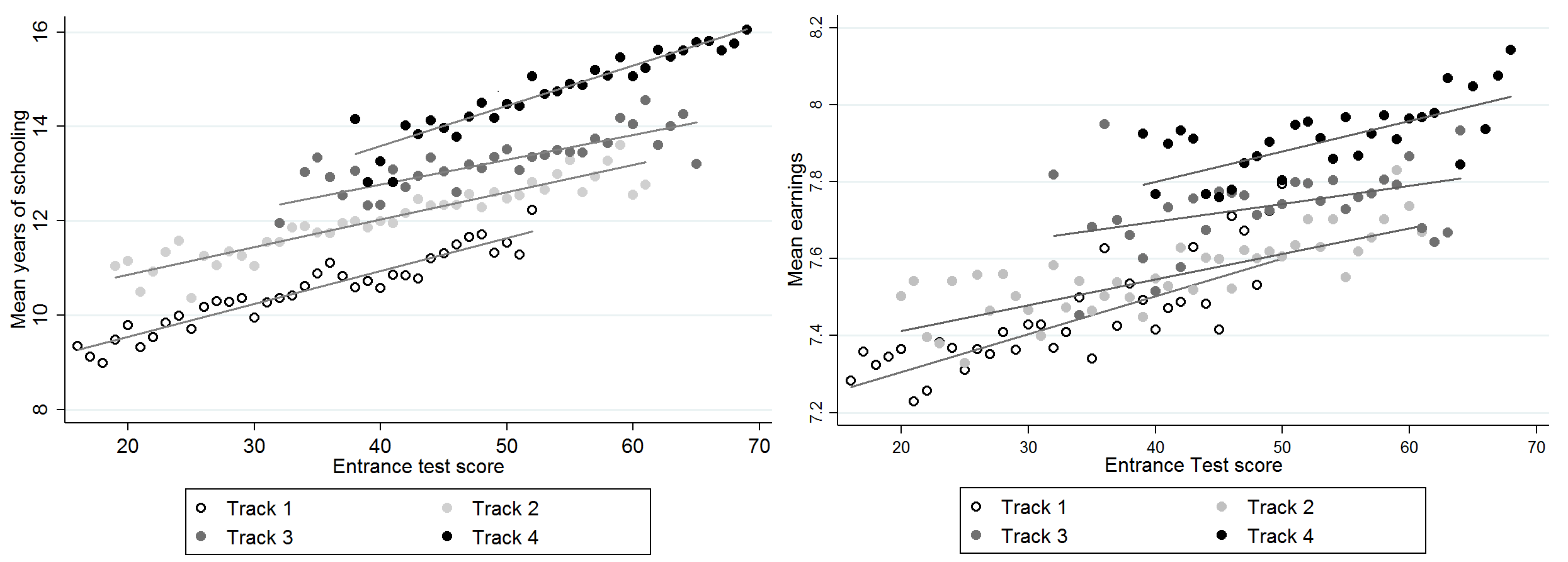

Supplement: S3 Fig — Mean years of schooling (left) and wages (right; in logs) for every test score on the Entrance Test and for every track, for cohort 1977. (TIF) [file pone.0215493.s003.tif]

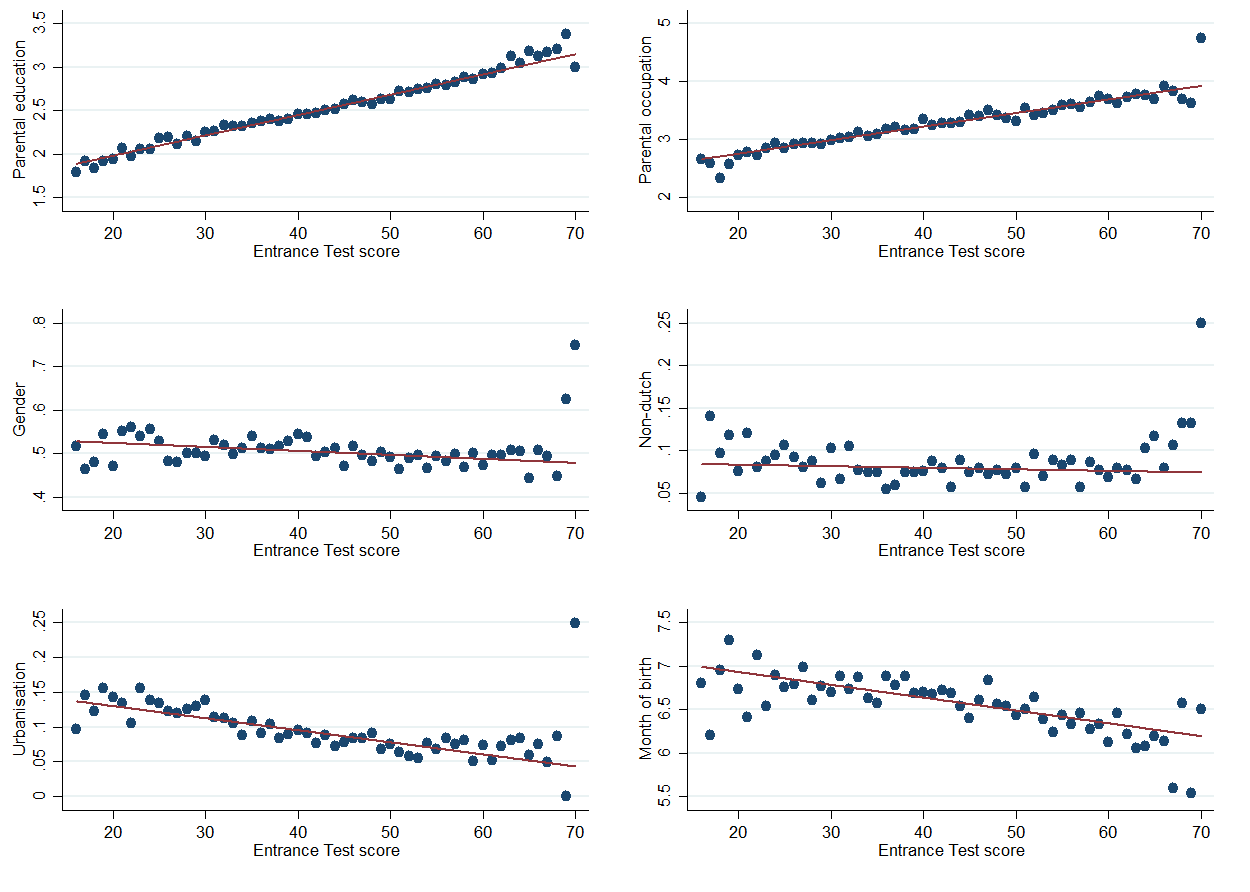

Supplement: S4 Fig — Mean values of all control variables for every score on the Entrance Test, for the 1977 cohort. Parental education and parental occupation are measured as continuous variables. (TIF) [file pone.0215493.s004.tif]

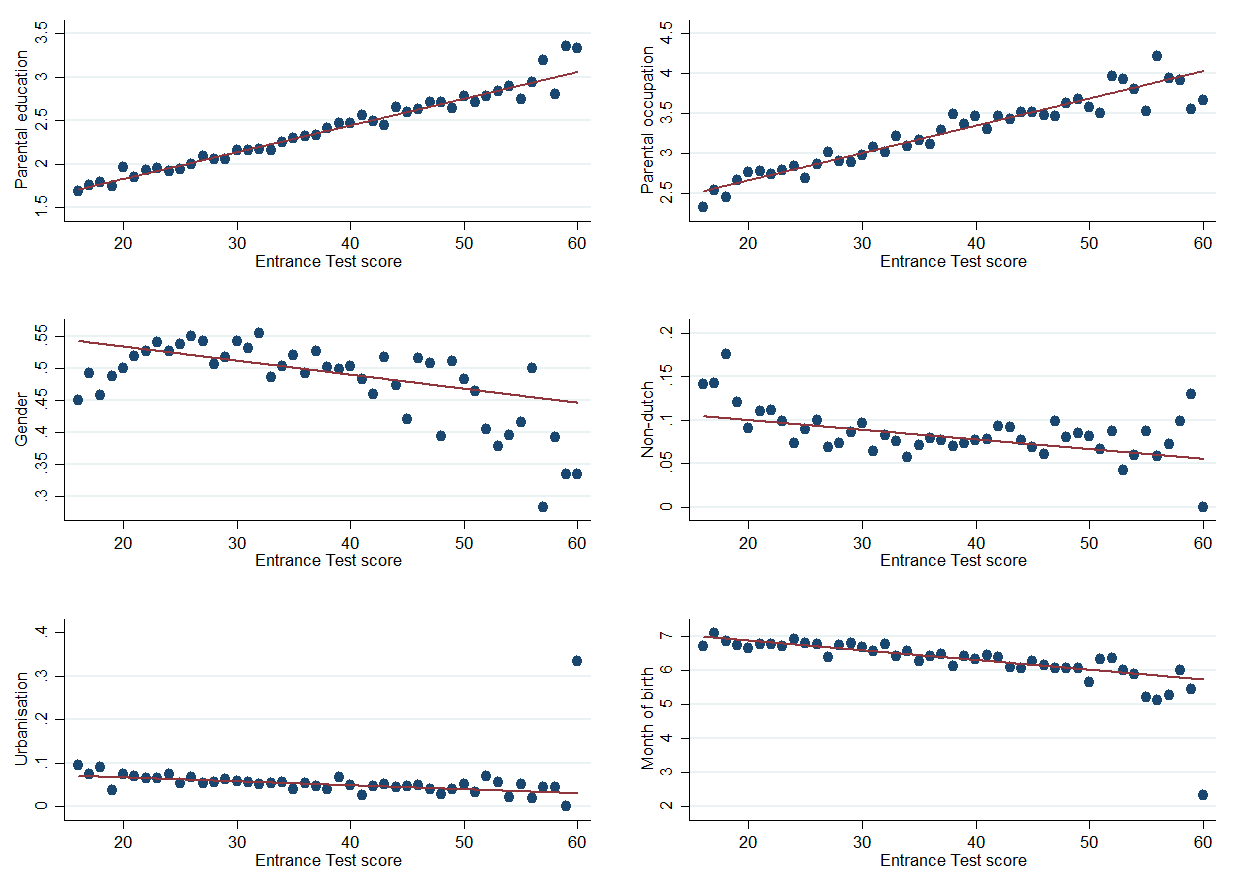

Supplement: S5 Fig — Mean values of all control variables for every score on the Entrance Test, for the 1983 cohort. Parental education and parental occupation are measured as continuous variables. (TIF) [file pone.0215493.s005.tif]

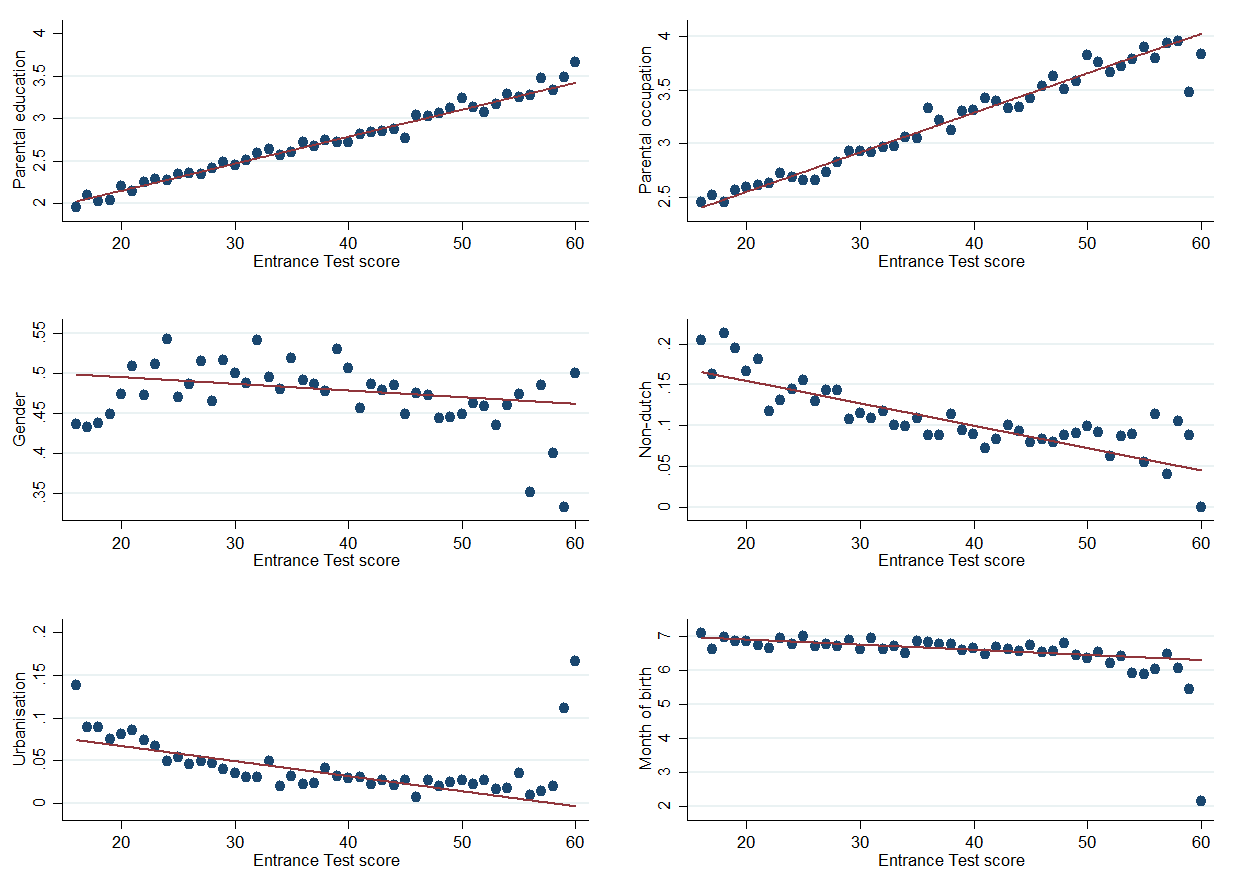

Supplement: S6 Fig — Mean values of all control variables for every score on the Entrance Test, for the 1989 cohort. Parental education and parental occupation are measured as continuous variables. (TIF) [file pone.0215493.s006.tif]

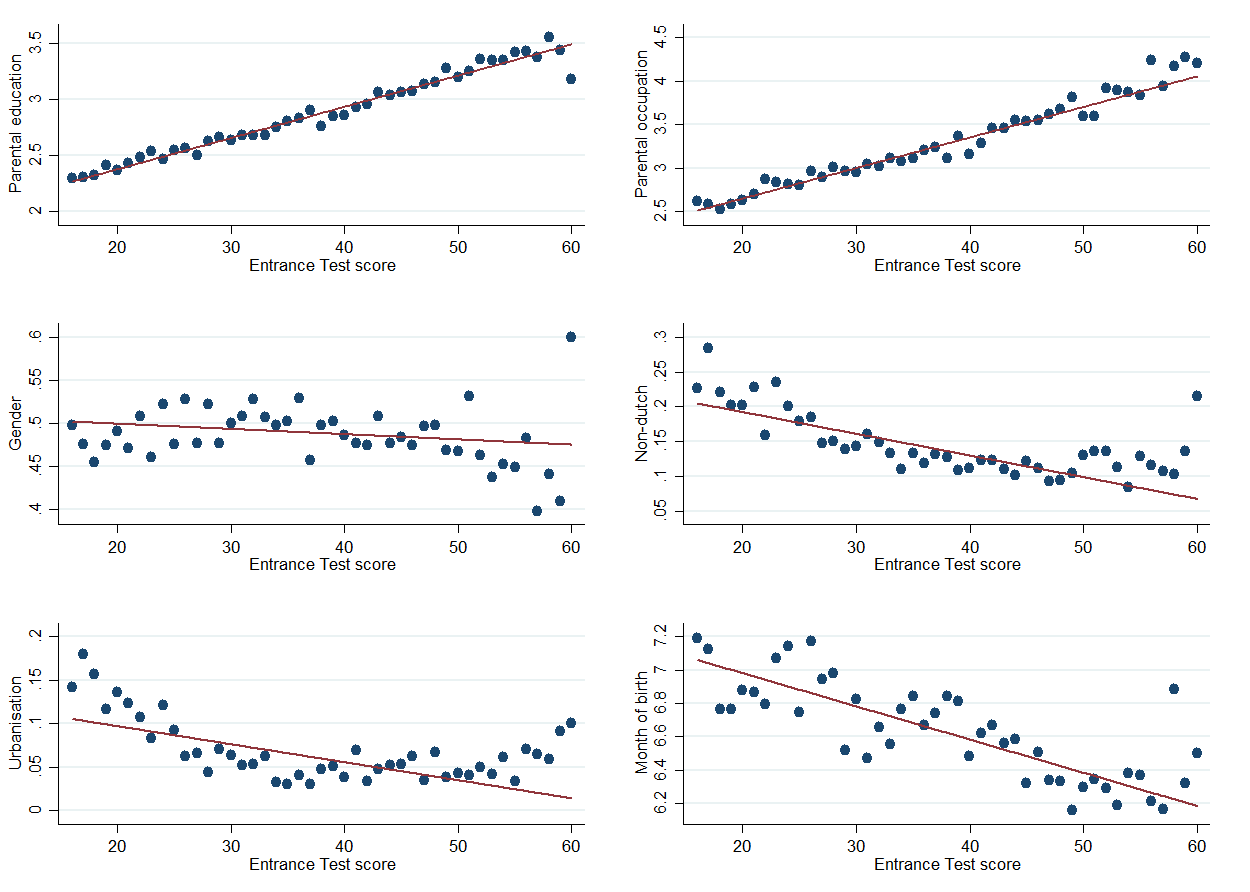

Supplement: S7 Fig — Mean values of all control variables for every score on the Entrance Test, for the 1993 cohort. Parental education and parental occupation are measured as continuous variables. (TIF) [file pone.0215493.s007.tif]

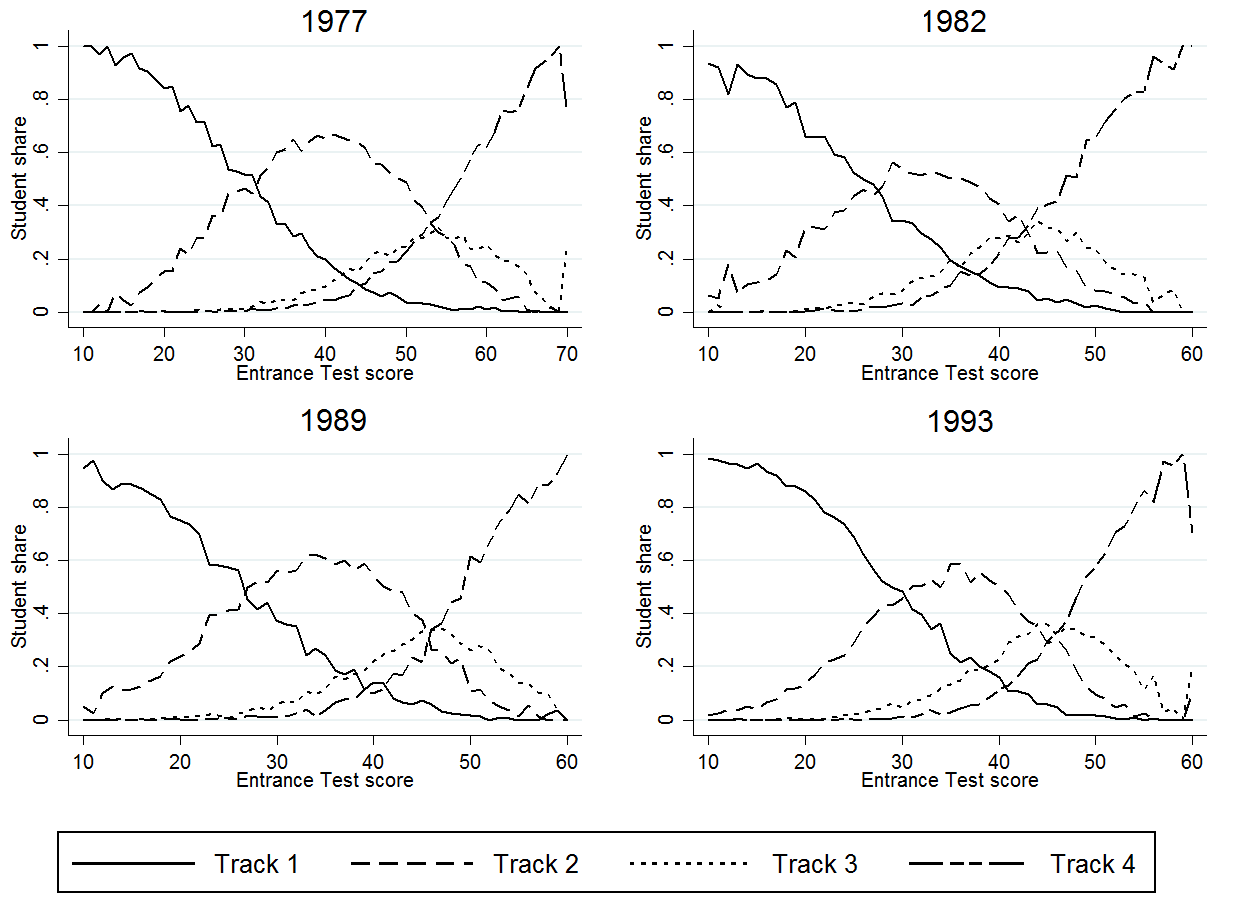

Supplement: S8 Fig — Shares of students assigned to a certain track for each score on the Entrance Test, across cohorts. (TIF) [file pone.0215493.s008.tif]

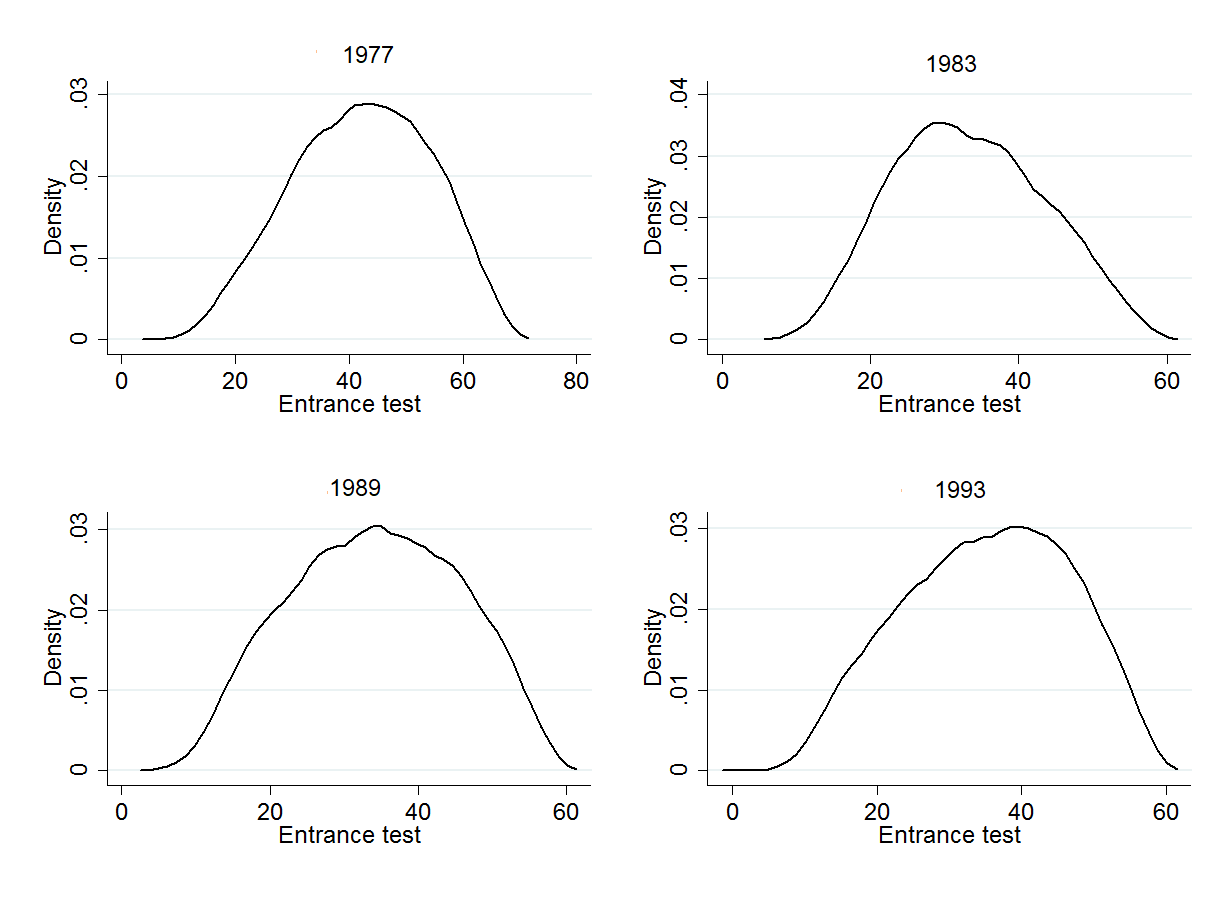

Supplement: S9 Fig — Density of the Entrance Test score, across cohorts. (TIF) [file pone.0215493.s009.tif]
