# Supplementary material for: The long-run effects of secondary school track assignment
Source: PLoS One. 2019 Oct 25;14(10):e0215493. doi: 10.1371/journal.pone.0215493 (PMC6814234; doi:10.1371/journal.pone.0215493)
Supplement: S1 Table — (PDF) [file pone.0215493.s010.pdf]

S1 Table. First stage results.

	T2 vs. T1		T3 vs. T2		T4 vs. T3	
	YoS	Wage	YoS	Wage	YoS	Wage
Panel A: Estimates						
1977 cohort	0.974***	0.963***	0.999***	1.01***	0.970***	0.971***
	(0.129)	(0.055)	(0.095)	(0.049)	(0.027)	(0.027)
1983 cohort	0.945***	0.936***	1.00***	1.04***	0.981***	0.977***
	(0.093)	(0.068)	(0.128)	(0.118)	(0.035)	(0.036)
1989 cohort	0.948***	0.930***	0.990***	1.00***	0.963***	0.955***
	(0.093)	(0.085)	(0.140)	(0.054)	(0.034)	(0.065)
1993 cohort	0.944***	-	0.982***	-	0.939***	-
	(0.105)		(0.088)		(0.097)	
Panel B: KP-stats						
1977 cohort	57.00	300.20	110.78	387.95	1325.61	1145.65
1983 cohort	102.07	178.53	61.15	67.99	765.10	655.89
1989 cohort	102.73	109.33	49.79	329.92	770.86	197.48
1993 cohort	80.70	-	125.39	-	93.60	-

Notes: *Significant at 10% level **Significant at 5% level ***Significant at 1% level
Panel A shows the first stage estimates for instrument w_i^M in Model (3). Standard errors are between parentheses and are robust and corrected for clustering at the school level. Panel B reports the Kleibergen-Paap F-statistic on the strength of the instrument.
