# Supplementary material for: The long-run effects of secondary school track assignment
Source: PLoS One. 2019 Oct 25;14(10):e0215493. doi: 10.1371/journal.pone.0215493 (PMC6814234; doi:10.1371/journal.pone.0215493)
Supplement: S2 Table — (PDF) [file pone.0215493.s011.pdf]

S2 Table. Sensitivity of results to bandwidth.

T2 vs. T1

	BL	LL-	LL+	LL++	UL-	UL- -	UL+	UL++
1977 YoS	1.76**	1.84***	1.87***	1.79**	2.81***	2.25**	1.51***	1.11**
	(0.706)	(0.694)	(0.729)	(0.743)	(0.942)	(1.01)	(0.445)	(0.323)
1983 YoS	1.17	1.64***	0.823	3.33*	2.63***	2.32*	2.15***	1.82***
	(0.752)	(0.636)	(1.06)	(1.98)	(0.938)	(1.36)	(0.642)	(0.621)
1977 wage	0.147***	0.148***	0.144***	0.135***	0.104**	0.243***	0.161***	0.158***
	(0.039)	(0.039	(0.039)	(0.044)	(0.049)	(0.072)	(0.035)	(0.035)
1983 wage	0.152***	0.138***	0.139**	0.186***	0.121**	0.133*	0.150***	-
	(0.051)	(0.050)	(0.062)	(0.082)	(0.056)	(0.073)	(0.050)	

T3 vs. T2

	BL	LL-	LL- -	LL+	LL++	UL-	UL- -	UL- - -
1977 YoS	0.204	-0.626	-0.633*	-0.935	0.134	0.330	1.05	1.13
	(0.605)	(0.440)	(0.344)	(0.992)	(1.35)	(0.614)	(0.642)	(0.807)
1983 YoS	0.010	-0.853	-1.37***	1.33	2.28	-0.326	-0.378	0.036
	(0.929)	(0.602)	(0.476)	(1.38)	(1.74)	(0.928)	(0.928)	(1.03)
1977 wage	-0.122***	-0.130***	-0.138***	-0.152***	-0.140**	-0.124***	-0.090**	-0.064
	(0.040)	(0.036)	(0.035)	(0.049)	(0.065)	(0.040)	(0.042)	(0.049)
1983 wage	-0.122	-0.098*	-0.110**	-0.189	-0.078	-0.123	-0.116	-0.099
	(0.086)	(0.057)	(0.045)	(0.154)	(0.178)	(0.086)	(0.087)	(0.090)

T4 vs. T3

	BL	LL- -	LL+	LL++	LL+++	UL-	UL- -	UL+
1977 YoS	1.00***	1.01***	1.14***	1.14***	1.25***	1.11***	0.592	0.966***
	(0.199)	(0.194)	(0.211)	(0.235)	(0.283)	(0.231)	(0.403)	(0.184)
1983 YoS	1.25***	1.02***	1.36***	0.692	0.509	1.11***	0.985**	-
	(0.339)	(0.271)	(0.461)	(0.739)	(1.19)	(0.361)	(0.446)	
1977 wage	0.071***	0.068***	0.079***	0.070**	0.084**	0.085**	0.099**	0.075***
	(0.026)	(0.026)	(0.028)	(0.032)	(0.038)	(0.033)	(0.049)	(0.024)
1983 wage	0.027	0.040	-0.016	-0.011	0.066	0.0083	0.050	-
	(0.034)	(0.027)	(0.046)	(0.069)	(0.104)	(0.036)	(0.045)	

Notes: *Significant at 10% level **Significant at 5% level ***Significant at 1% level
The table shows the sensitivity of the estimates from Table 3 in the main article to changes in the bandwidth. The first entry shows the result for the baseline bandwidth (BL). Other entries show estimates for changes in the upper limit (UL) and lower limit (LL) of the bandwidth. Bandwidths change with intervals of 5. For example, for the first row the bandwidths are: [10-47], [5-47], [15-47], [20-47], [10-42], [10-37], [10-52], [10-57]. Open entries imply that the end of the bandwidth is already reached. See S3 Table for a full overview of all bandwidths in this exercise. YoS = Years of Schooling. Standard errors are between parentheses and are robust and corrected for clustering at the school level.
