# Supplementary material for: The long-run effects of secondary school track assignment
Source: PLoS One. 2019 Oct 25;14(10):e0215493. doi: 10.1371/journal.pone.0215493 (PMC6814234; doi:10.1371/journal.pone.0215493)
Supplement: S3 Table — (PDF) [file pone.0215493.s012.pdf]

S3 Table. Bandwidths for sensitivity exercise.

T2 vs. T1

	BL	LL-	LL+	LL++	UL-	UL- -	UL+	UL++
1977 YoS	[10-47]	[5-47]	[15-47]	[20-47]	[10-42]	[10-37]	[10-52]	[10-57]
N	15,433	15,447	15,284	14,634	12,230	8,866	18,019	19,690
1983 YoS	[22-48]	[17-48]	[27-48]	[32-48]	[22-43]	[22-38]	[22-53]	[22-58]
N	9,758	11,050	7,626	5,184	8,952	7,505	10,012	10,048
1977 wage	[12-59]	[7-59]	[17-59]	[22-59]	[12-54]	[12-49]	[12-64]	[12-69]
N	15,969	15,993	15,743	15,096	14,915	13,025	16,330	16,364
1983 wage	[15-55]	[10-55]	[20-55]	[25-55]	[15-50]	[15-45]	[15-60]	-
N	9,697	9,883	8,954	7,498	9,576	9,081	9,704	-

T3 vs. T2

	BL	LL-	LL- -	LL+	LL++	UL-	UL- -	UL- - -
1977 YoS	[33-70]	[28-70]	[23-70]	[38-70]	[43-70]	[33-65]	[33-60]	[33-55]
N	14,749	17,303	19,005	11,635	8,271	14,731	14,432	13,377
1983 YoS	[26-60]	[21-60]	[16-60]	[31-60]	[36-60]	[26-55]	[26-50]	[26-45]
N	8,397	10,389	11,498	5,963	3,700	8,390	8,254	7,689
1977 wage	[20-70]	[15-70]	[10-70]	[25-70]	[30-70]	[20-65]	[20-60]	[20-55]
N	15,809	16,266	16,379	14,864	13,297	15,794	15,543	14,630
1983 wage	[25-60]	[20-60]	[15-60]	[30-60]	[35-60]	[25-55]	[25-50]	[25-45]
N	7,505	8,961	9,704	5,562	3,607	7,498	7,377	6,882

T4 vs. T3

	BL	LL- -	LL+	LL++	LL+++	UL-	UL- -	UL+
1977 YoS	[15-64]	[5-64]	[20-64]	[25-64]	[30-64]	[15-59]	[15-54]	[15-69]
N	24,448	24,611	23,798	22,508	20,470	23,023	20,506	24,898
								,
1983 YoS	[21-60]	[11-60]	[26-60]	[31-60]	[36-60]	[21-55]	[21-50]	-
N	12,811	14,288	10,806	8,316	5,892	12,676	12,069	-
1977 wage	[15-64]	[5-64]	[20-64]	[25-64]	[30-64]	[15-59]	[15-54]	[15-69]
N	19,772	19,896	19,315	18,369	16,797	18,543	16,394	20,161
1983 wage	[21-60]	[11-60]	[26-60]	[31-60]	[36-60]	[21-55]	[21-50]	-
N	10,926	12,068	9,335	7,298	5,237	10,800	10,254	-

Notes: The table shows the bandwidths and corresponding sample sizes used in the sensitivity exercise presented in S2 Table.
