# Supplementary material for: The long-run effects of secondary school track assignment
Source: PLoS One. 2019 Oct 25;14(10):e0215493. doi: 10.1371/journal.pone.0215493 (PMC6814234; doi:10.1371/journal.pone.0215493)
Supplement: S4 Table — (PDF) [file pone.0215493.s013.pdf]

S4 Table. Main approach versus RDD for 9th grade achievement (1999).

	T3 vs. T1/T2			T4 vs. T3		
	Language	Math	PS	Language	Math	PS
Main approach	0.299**	0.461*	-0.0024	0.405*	0.406*	0.080
	(0.132)	(0.237)	(0.140)	(0.209)	(0.237)	(0.082)
RDD	0.341***	0.593***	0.144	0.529**	0.240	0.071
	(0.166)	(0.210)	(0.190)	(0.249)	(0.286)	(0.093)

Notes: *Significant at 10% level **Significant at 5% level ***Significant at 1% level
The table shows the estimates of the effect of track assignment for 9th grade achievement for the 1999 cohort, for both the main approach (Table 4 in the main article) and a traditional Regression Discontinuity Design (RDD). The RDD approach uses a dichotomous instrument for whether the score on the CITO test is above or below the official threshold. See Table 4 in the main article for bandwidths and sample sizes. Standard errors are between parentheses and are robust and corrected for clustering at the school level.
