# Supplementary material for: The long-run effects of secondary school track assignment
Source: PLoS One. 2019 Oct 25;14(10):e0215493. doi: 10.1371/journal.pone.0215493 (PMC6814234; doi:10.1371/journal.pone.0215493)
Supplement: S5 Table — (PDF) [file pone.0215493.s014.pdf]

S5 Table. Effect of track assignment on study choice.

	T2 vs. T1			T3 vs. T2			T4 vs. T3		
	S1	S2	S3	S1	S2	S3	S1	S2	S3
1977 cohort	0.0024	0.097***	0.080***	0.119*	-0.095	-0.057	0.055***	0.0026	0.016
	(0.033)	(0.035)	(0.029)	(0.070)	(0.072)	(0.064)	(0.012)	(0.016)	(0.013)
Bandwidth	[12-59]	[12-59]	[12-59]	[33-70]	[33-70]	[33-70]	[15-64]	[15-64]	[15-64]
Mean wage	2208	3489	3838	2567	3710	4029	3482	4827	4724
N		20,016			14,749			24,448	
1982 cohort	0.205**	0.125	0.079	0.045	-0.150***	0.0073	0.091***	-0.013	0.025
	(0.099)	(0.091)	(0.081)	(0.088)	(0.054)	(0.048)	(0.025)	(0.038)	(0.067)
Bandwidth	[15-45]	[15-45]	[15-45]	[15-50]	[15-50]	[15-50]	[21-60]	[21-60]	[21-60]
Mean wage	1901	3008	3341	2253	3294	3571	3094	4458	4169
N		10,923			11,488			12,811	
1989 cohort	-0.081	0.078	-0.052	0.106*	-0.087	0.020	0.116***	-0.059	-0.011
	(0.084)	(0.088)	(0.072)	(0.065)	(0.065)	(0.057)	(0.047)	(0.061)	(0.040)
Bandwidth	[19-45]	[19-45]	[19-45]	[25-60]	[25-60]	[25-60]	[35-55]	[35-55]	[35-55]
Mean wage	1932	2527	2763	2158	2817	2990	2705	3506	3353
N		11,779			11,004			14,847	

Notes: *Significant at 10% level **Significant at 5% level ***Significant at 1% level
The table shows the estimates of the effect of track assignment on field of study. 'S1' combines humanities, health and leisure; 'S2' combines economics and law, 'S3' combines math and technical studies. Mean 2007 wages are reported per study field, for those in the two relevant tracks and with employment in 2007. Standard errors are between parentheses and are robust and corrected for clustering at the school level. Bandwidths are reported between brackets.
