# Supplementary material for: The long-run effects of secondary school track assignment
Source: PLoS One. 2019 Oct 25;14(10):e0215493. doi: 10.1371/journal.pone.0215493 (PMC6814234; doi:10.1371/journal.pone.0215493)
Supplement: S6 Table — (PDF) [file pone.0215493.s015.pdf]

S6 Table. Logistic approach.

	T2 vs. T1		T2 vs. T1		T4 vs. T3	
	Main	Log.	Main	Log.	Main	Log.
1977 YoS	1.76**	1.08	0.204	0.334	1.00***	0.997***
	(0.706)	(0.750)	(0.605)	(0.681)	(0.199)	(0.199)
1983 YoS	1.17	1.30*	0.010	-1.65	1.25***	1.33***
	(0.753)	(0.777)	(0.929)	(1.29)	(0.339)	(0.347)
1989 YoS	1.04	1.45	-0.028	-1.34	1.54***	1.47***
	(0.729)	(1.23)	(0.981)	(1.23)	(0.273)	(0.272)
1993 YoS	1.19*	0.982	0.139	0.292	2.06*	2.59*
	(0.716)	(0.773)	(0.710)	(0.756)	(1.06)	(1.53)
1977 wage	0.147***	0.125***	-0.122***	-0.138***	0.071***	0.071***
	(0.039)	(0.041)	(0.040)	(0.042)	(0.027)	(0.027)
1983 wage	0.152***	0.147***	-0.122	-0.163	0.027	0.029
	(0.051)	(0.066)	(0.086)	(0.105)	(0.034)	(0.035)
1989 wage	0.088**	0.094**	-0.053*	-0.062**	0.013	-0.020
	(0.038)	(0.043)	(0.029)	(0.030)	(0.021)	(0.045)

Notes: *Significant at 10% level **Significant at 5% level ***Significant at 1% level
The table shows the estimates of the effect of track assignment when Model (3) is estimated with an alternative instrument. The instrument predicts the treatment probability from the Entrance test score, assuming a logistic relation. As such, any noise in the conditional mean instrument is smoothed out, compared to the main estimation approach. Bandwidths and sample sizes are the same as in the main model (Table 3 in the main article). Standard errors are between parentheses and are robust and corrected for clustering at the school level.
