# Supplementary material for: The long-run effects of secondary school track assignment
Source: PLoS One. 2019 Oct 25;14(10):e0215493. doi: 10.1371/journal.pone.0215493 (PMC6814234; doi:10.1371/journal.pone.0215493)
Supplement: S7 Table — (PDF) [file pone.0215493.s016.pdf]

S7 Table. Placebo tests.

	T2 vs. T1	T3 vs. T2	T4 vs. T3
1977 YoS	0.330	-0.137	0.0047
	(0.285)	(0.191)	(0.070)
1983 YoS	0.365	0.085	-0.110
	(0.250)	(0.329)	(0.126)
1989 YoS	0.321	0.032	0.236
	(0.335)	(0.451)	(0.162)
1993 YoS	0.434	-0.257	-0.348
	(0.368)	(0.287)	(0.427)
1977 wage	0.015	-0.016	0.029
	(0.024)	(0.024)	(0.019)
1983 wage	-0.038*	-0.0033	0.0029
	(0.023)	(0.040)	(0.014)
1989 wage	-0.0084	0.0084	0.011
	(0.010)	(0.0083)	(0.0081)

Notes: *Significant at 10% level **Significant at 5% level ***Significant at 1% level
The table shows the estimates for placebo tests that use the constructed vector of controls (Fig 7 of the main article) as outcome variables in Model (3). Bandwidths and sample sizes are the same as in the main estimation (Table 3 of the main article). Standard errors are between parentheses and are robust and corrected for clustering at the school level.
