# Supplementary material for: The long-run effects of secondary school track assignment
Source: PLoS One. 2019 Oct 25;14(10):e0215493. doi: 10.1371/journal.pone.0215493 (PMC6814234; doi:10.1371/journal.pone.0215493)
Supplement: S8 Table — (PDF) [file pone.0215493.s017.pdf]

S8 Table. Effect of track assignment: different sample compositions.

	T2 vs. T1			T3 vs. T2			T4 vs. T3		
	Main	A1	A2	Main	A1	A2	Main	A1	A2
1977 YoS	1.76***	1.53*	1.38***	0.204	0.521	0.078	1.00***	1.91***	2.34**
	(0.706)	(0.853)	(0.313)	(0.605)	(0.721)	(0.467)	(0.199)	(0.263)	(0.942)
BW	[10-47]	[10-44]	[10-60]	[33-70]	[10-46]	[25-70]	[15-64]	[25-70]	[10-70]
N	15,433	13,893	16,913	14,749	9,582	14,087	24,448	18,636	8,060
1983 YoS	1.17	1.83**	1.58**	0.010	0.161	0.137	1.25***	1.89***	0.904
	(0.753)	(0.811)	(0.663)	(0.929)	(1.06)	(1.14)	(0.339)	(0.403)	(1.19)
BW	[22-48]	[10-42]	[20-50]	[26-60]	[26-55]	[26-60]	[21-60]	[10-55]	[10-60]
N	9,758	11,368	8,592	8,397	8,494	6,220	12,811	9,586	4,464
1989 YoS	1.04	0.775	1.67**	-0.028	0.631	-0.176	1.54***	1.75***	1.34
	(0.729)	(0.721)	(0.775)	(0.981)	(0.653)	(1.09)	(0.273)	(0.279)	(0.972)
BW	[5-40]	[5-40]	[5-40]	[32-60]	[10-45]	[32-60]	[20-53]	[10-60]	[30-60]
N	11,560	11,879	10,732	7,725	8,545	6,463	14,847	11,729	4,798
1993 YoS	1.19*	1.35*	1.09	0.139	0.841	-0.368	2.06*	1.46***	1.00***
	(0.716)	(0.706)	(0.780)	(0.170)	(0.632)	(0.866)	(1.06)	(0.286)	(0.229)
BW	[10-40]	[10-40]	[10-40]	[28-60]	[10-45]	[28-60]	[10-60]	[20-60]	[20-60]
N	11,558	11,831	10,629	10,384	8,139	7,883	6,014	11,885	16,631
1977 wage	0.147***	0.106*	0.180***	-0.122***	-0.102	-0.099	0.071**	0.110***	0.178*
	(0.039)	(0.062)	(0.059)	(0.040)	(0.130)	(0.061)	(0.027)	(0.034)	(0.105)
BW	[12-59]	[10-50]	[26-60]	[20-70]	[10-44]	[25-70]	[15-64]	[25-66]	[10-65]
N	15,969	14,401	11,584	15,809	6,631	11,532	19,772	15,268	6,621
1983 wage	0.152***	0.103	0.072	-0.122	-0.087	-0.098	0.027	0.053	-0.0059
	(0.051)	(0.085)	(0.078)	(0.086)	(0.137)	(0.109)	(0.034)	(0.043)	(0.104)
BW	[15-55]	[10-41]	[20-50]	[25-60]	[28-54]	[25-60]	[21-60]	[21-60]	[10-55]
N	9,697	9,161	7,104	7,505	6,981	5,590	10,926	8,253	3,889
1989 wage	0.088	0.074	0.038	-0.053*	0.085	-0.053	0.013	0.0096	0.0043
	(0.038)	(0.070)	(0.071)	(0.029)	(0.078)	(0.038)	(0.021)	(0.025)	(0.062)
BW	[19-55]	[10-39]	[5-40]	[20-60]	[10-53]	[20-60]	[20-53]	[20-53]	[10-53]
N	11,828	9,927	9,397	11,591	10,175	8,082	13,445	9,937	4,037

Notes: *Significant at 10% level **Significant at 5% level ***Significant at 1% level
The table shows the estimates of the effect of track assignment for different sample compositions. For T2 vs. T1, results are shown for inclusion of the lowest three tracks (Main), all four tracks (A1) and the lowest two tracks (A2). For T3 vs. T2, results are shown for the lowest three tracks (Main), the highest three tracks (A1) and the two middle tracks (A2). For T4 vs. T3, results are shown for all four tracks (Main), the highest three tracks (A1) and the highest two tracks (A2). For T4 vs. T3 with respect to 1993 YoS, the main model only includes the two tracks at the margin, and A1 and A2 show results for the highest three tracks and all four tracks, respectively. Standard errors are between parentheses and are robust and corrected for clustering at the school level. Optimal bandwidths are restimated for the alternative samples, and are provided between brackets.
